# Pediatric hypersensitivity pneumonitis: literature update and proposal of a diagnostic algorithm

**DOI:** 10.1186/s13052-022-01239-0

**Published:** 2022-03-28

**Authors:** Carla Mastrorilli, Luca Pecoraro, Stefania Arasi, Simona Barni, Lucia Caminiti, Riccardo Castagnoli, Mattia Giovannini, Lucia Liotti, Francesca Mori, Francesca Saretta, Gian Luigi Marseglia, Elio Novembre, Rare Allergic Diseases Commission of the Italian Society of Pediatric Allergy and Immunology, Carla Mastrorilli, Luca Pecoraro, Stefania Arasi, Simona Barni, Lucia Caminiti, Riccardo Castagnoli, Mattia Giovannini, Lucia Liotti, Francesca Mori, Francesca Saretta, Gian Luigi Marseglia, Elio Novembre

**Affiliations:** 1 Pediatric and Emergency Unit, University Hospital Consortium Corporation Polyclinic of Bari, Pediatric Hospital Giovanni XXIII, Bari, Italy; 2 Department of Medicine, University of Verona, Policlinico GB Rossi, Verona, Italy; 3 Pediatric Unit, ASST Mantua, Mantua, Italy; 4 Translational Research in Pediatric Specialties Area, Division of Allergy, Bambino Gesù Children’s Hospital IRCCS, Rome, Italy; 5 Allergy Unit, Department of Pediatrics, Meyer Children’s University Hospital, Florence, Italy; 6 Department of Human Pathology in Adult and Development Age “Gaetano Barresi”, Allergy Unit, Department of Pediatrics, AOU Policlinico Gaetano Martino, Messina, Italy; 7 Department of Pediatrics, Pediatric Clinic, Fondazione IRCCS Policlinico San Matteo, University of Pavia, Pavia, Italy; 8 Pediatric Unit, Senigallia Hospital, Senigallia, Italy; 9 Pediatric Department, Latisana-Palmanova Hospital, Azienda Sanitaria Universitaria Friuli Centrale, Udine, Italy

**Keywords:** Children, Cough, Dyspnea, Extrinsic allergic alveolitis, Hypersensitivity pneumonitis, Interstitial pneumonia, Pediatric

## Abstract

Hypersensitivity pneumonitis (HP) is a rare disease in childhood with the prevalence of 4 cases per 1 million children and an incidence of 2 cases per year. The average age of diagnosis at pediatric age is approximately 10 years. The pathogenesis of HP is characterized by an immunological reaction caused by recurrent exposure to triggering environmental agents (mostly bird antigens in children). The clinical picture of HP is complex and variable in children, often presenting in subacute forms with cough and exertion dyspnea. A diagnosis of HP should be considered in patients with an identified exposure to a triggering antigen, respiratory symptoms, and radiologic signs of interstitial lung disease. Blood tests and pulmonary function tests (PFT) support the diagnosis. Bronchoscopy (with bronchoalveolar lavage and tissue biopsy) may be needed in unclear cases. Antigen provocation test is rarely required. Of note, the persistence of symptoms despite various treatment regimens may support HP diagnosis. The avoidance of single/multiple triggers is crucial for effective treatment. No evidence- based guidelines for treatment are available; in particular, the role of systemic glucocorticoids in children is unclear. With adequate antigen avoidance, the prognosis in children with HP is generally favorable.

## Introduction

Hypersensitivity pneumonitis (HP), also named extrinsic allergic alveolitis (EAA) in Europe, is the most frequent chronic interstitial lung disease in children. HP is an immune-mediated inflammatory condition. It involves the distal portions of the lungs and is caused by massive and/or repeated exposure to various environmental antigens. HP usually presents in the adult population. However, it is also reported in children; in particular, 8% of patients are younger than 15 years of age at the diagnosis [1] and often goes unrecognized. Specifically, childhood HP is often associated with exposure to antigens in the home environment or related to specific hobbies [2]. HP belongs to the group of childhood interstitial lung disease (chILD), a heterogeneous category of respiratory disorders that are primarily chronic and impair the respiratory function of the lungs [3]. This review aims to update the current evidence on HP in children. Moreover, it is intended as a practical guide for clinicians treating children with HP. In this context, we reviewed the topic and proposed the first pediatric diagnostic algorithm related to this illness.

## Research strategies and literature analysis

We carried out a non-systematic review including the most relevant studies on “Hypersensitivity pneumonitis” in databases including PubMed and the Cochrane Library from January 1932 until May 2021. Manuscripts were selected among randomized controlled trials, case reports, reviews, systematic reviews, cohort and case-control studies, and observational studies. Articles in non-English language were excluded. The terms searched for were “Hypersensitivity pneumonitis” [all fields]; “Hypersensitivity pneumonitis” and “children” [all fields]; “pediatric Hypersensitivity pneumonitis” [all fields].

## Epidemiology

Although HP is the most frequent chronic interstitial lung disease in children, it is often underdiagnosed in this population because it is considered an adulthood illness [2]. Of note, the first case of HP in an adult was described by Campbell et al. in 1932 [4], while the first pediatric case was reported only in 1967 [5]. By then, some pediatric HP cases have been described in the literature [1, 6]. However, the lack of uniform diagnostic criteria makes it difficult to define the exact frequency of the disease in childhood.

The only attempt to define the epidemiology of HP was conducted by Buchvald et al. [7], who reported a prevalence of 4 cases/1 million children and an incidence of 2 cases/year. Regarding gender, 95% of the cases were males [8]. Moreover, HP is most frequently diagnosed in children aged approximately 10 years [7]. The disease is related to several factors, such as the amount of allergen inhaled, the nature of the antigen, the duration of exposure, and the host immune response [9]. In addition, genetic factors may play an important role as 25% of children have a positive family history [9]. Specifically, HLA loci involved are HLA-DR7, HLA-B8, and HLA-DQw3 [9–11]. However, shared exposure to the same triggering antigens should be considered when more family members present the disease.

It has been reported that HP represents almost 50% of chILD [12, 13]. Of note, chILD occurs more frequently at a younger age and in boys [3, 14–16].

## Pathogenesis

The pathogenesis of HP in children is principally related to two factors; the type of allergen inhaled and the host immune response [9]. To the best of our knowledge, there are no experimental studies about the pathogenesis of HP conducted on children. However, the pathophysiological basis of this illness seems to be the same as the adult age.

### Etiologic agents

Some specific groups of allergens are related to the pathogenesis of HP (Table 1) [17, 18]. In order to trigger the disease, the antigen should be able to enter the small airways; thus, its size must be within the respirable range (< 5 mm) [19]. About the pediatric population, antigens causing HP are often related to specific hobbies and are found in the domestic environment: avian, fungal, and mold or various inorganic antigens, such as inhaled paints, plastics, wax, and talcum [7, 10, 20]. About microorganisms, thermophilic actinomycetes are present in the farms and represent the most studied antigens. These antigens are related to the classic farmer’s lung disease [21]. Other types of bacteria and fungi involved in the pathogenesis of HP are Aspergillus sp., Candida sp., Cephalosporium, *Aureobasidium pullulans*, *Naegleria gruberi*, *Acanthamoeba polyphagia*, *Acanthamoeba castellani*, Bacillus spp., Trichosporon sp., *Cryptococcus albidus*, *Mycobacterium avium* complex [2, 22, 23]. About plant proteins, Soybean, Coffee, and Lycoperdon spp. have been described [2]. Isocyanates paints, anhydrides, and pyrethrum are low molecular weight chemicals contained in plastics and insecticides [2]. HP cases caused by e-cigarette use have been recently reported [24, 25]. In addition, a case of childhood HP related to secondhand smoke from an electronic cigarette has been described [26].

**Table 1:** Groups of allergens related to the pathogenesis of HP in children

Environmental source class	Representative pathogens
Bacteria and mycobacteria	Thermophilic actinomycetes, *Mycobacterium avium complex*
Fungi	*Aspergillus* spp., *Alternaria* spp., *Penicillium* spp., *Trichosporon* spp.
Animal-derived proteins	Bird allergens, animal fur, cow’s milk
Plant proteins	Grain proteins, tea plants, coffee-bean dust
Chemicals	Plastics (e.g. isocyanates, anhydrides), detergents, pesticides (e.g. pyrethrum), e-cigarette liquids

A peculiar form of HP is represented by Heiner’s syndrome, caused by the intake of cow’s milk (CM). Sixty-one cases have been published in the literature, all in the pediatric age [27]. It is characterized by recurrent respiratory symptoms with pulmonary infiltrates at the chest radiography, poor growth, gastrointestinal symptoms, iron deficiency anemia, and occasionally pulmonary hemosiderosis. Serum precipitins for CM have been detected in several cases; however, they are not pathognomonic. The removal of CM from the diet resulted in almost complete resolution of the symptoms after a few days/weeks. Moreover, the reintroduction of milk caused a reoccurrence of the disease. Overall, this syndrome is so rare that it can be almost defined as anecdotal [27].

### The host immune response

HP is a consequence of an immunological reaction caused by recurrent exposure to environmental agents [28]. This exposure can occur in the workplace or at home. In addition, it can be related to hobbies or sometimes to an environment frequently visited by the patient. In general terms, HP trigger is found only in 40% of the cases [29]. Moreover, HP can be also induced by multiple allergens [28]. However, an individual genetic predisposition is considered fundamental to develop HP [28]. The interaction between environmental allergens and the immune system in genetically predisposed individuals generates an inflammatory state of alveoli, terminal bronchioli, and lung interstitium [30]. Both innate and adaptive immune responses contribute to the development of HP [30]. Specifically, the aberrant immune response leads to an exaggerated inflammatory reaction in the lungs [31]. The pathophysiological features of the interaction between allergens and the immune system are expressed differently in acute and chronic HP and lung fibrosis, which represents the last step of this illness [32]. Acute HP is characterized by high titers of antigen-specific precipitating IgG in the serum. The interaction between allergens and the immune system leads to the formation of immune complexes, with consequent lung inflammation [32].

On the other hand, an exaggerated T cell-mediated response characterizes chronic HP. Specifically, the activation of cell-mediated immune response leads to increase migration, local proliferation, and decreased apoptosis of T-cells in the bronchial and lung environment. It results in the characteristic T-lymphocytic alveolitis [33, 34]. The Th1 immune response is over-expressed, and the transcription factor STAT-4 and t-bet, in association with IL-12 and IFN-γ, seem to be involved in this process [33, 34] along with Th17-cells (IL-17A, IL-22) [35]. Lung fibrosis is characterized by a Th2 immune response with increased CD4+ T-cells and the CD4+/CD8+ ratio [36]. The involvement of Th17 cells may promote collagen deposition in the lung in response to chronic exposure to HP antigens [37].

### Clinical presentation and disease classifications

The clinical presentation of HP is complex and variable. It is dependent on the type, intensity, and duration of exposure to the causative agent, the susceptibility of the host, and the dysregulation of the immune system. Several clinical classifications of HP have succeeded over time. The classic classification is related to the division of HP into three categories according to the duration of the disease: acute, subacute, and chronic [38]. This type of grouping was not adequate because of its little prognostic value, the risk of overlapping between these three forms, and the new evidence arising from the analysis of bronchoalveolar lavage and imaging [28]. Vasakova et al. [28] recently proposed a classification of HP based on clinical–radiological–pathological correlation: acute/inflammatory HP and chronic/fibrotic HP. Specifically, acute/inflammatory HP is denoted by a disease lasting < 6 months and often reversible. In this form, symptoms are characterized by dyspnea, cough, and, less commonly, wheezing (5%); weight loss can be present [10]. An acute exacerbation has been defined as a meaningful deterioration of the child’s respiratory condition that needs a change in routine management [39]. Clemens et al. proposed clinical criteria defining acute exacerbations in chILD that can also be endorsed for HP [39] (Table 2). The specific signs on auscultation are represented by crackles and sometimes signs of bronchial obstruction [10].

**Table 2:** Acute exacerbation of HP in children (modified from [39])

Criteria *(>* *2 are needed for the definition of acute exacerbation*)	• Increase in respiratory rate > 20% from baseline • Onset or increase of dyspnea • Newly developing or increased abnormalities on chest imaging • Onset/increase of oxygen demand to achieve baseline saturation (at rest and/or during exercise) • Need for a supplementary level of ventilatory support (in addition to oxygen) • Decrease in vital capacity at spirometry in children able to perform the tests (> 10%) from baseline • Reduced exercise tolerance in children able to perform the tests (including desaturation)

On the other hand, chronic/fibrotic HP is characterized by > 6 months duration and the presence of fibrotic changes at high-resolution computed tomography (HRCT) images or histology [28]. Clinical manifestations include gradual respiratory failure with chronic dry cough and weight loss. Crackles on auscultation are present [10, 40]. Digital clubbing has been found in 13-31% of cases [10], suggesting that HP has been documented in the late stage.

However, acute/inflammatory HP and chronic/fibrotic HP do not represent two distinct entities in all cases. In the French cohort [41], the most frequent clinical presentation was the subacute form. The most frequent symptoms in the French and Danish cohorts [7] were exertion dyspnea and dry cough. Sometimes, HP can present through intermittent flares, with radiologic and/or histopathologic signs of combined active inflammatory and chronic changes [28].

The intensity and duration of exposure to the triggering factor determine the clinical course of the disease [17]. In most cases, children heal entirely after the avoidance of the triggering factor [10]. However, patients with well-defined HP without an identifiable trigger tend to have a chronic course (cryptogenic HP) [28, 42].

## Diagnosis

Currently, there are no distinct diagnostic criteria for hypersensitivity pneumonitis in the pediatric population compared to adults.

**Fig. 1:**
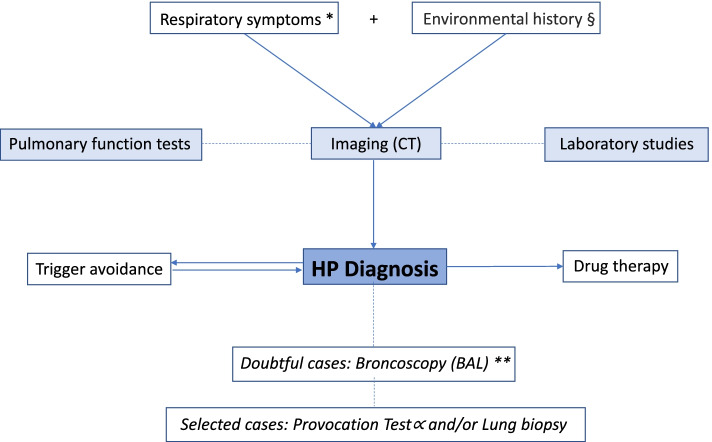
Diagnostic algorithm of childhood HP. Comments:^*^Respiratory symptoms: the clinical course can be chronic with dry cough and exertion dyspnoea in children. Usually, before diagnosis, children are treated as asthmatic without significant clinical improvement [7]. ^§^Environmental history: bird breeding in almost all pediatric cases [41]. ^**^in the presence of known exposure history to trigger typical HRCT and response to treatment, BAL is not necessary [29]

The diagnosis of HP requires a combination of respiratory signs and symptoms and characteristic radiologic features of diffuse lung disease. In 2016, the European Academy of Allergy and Clinical Immunology (EAACI) introduced the occupational hypersensitivity pneumonitis diagnostic criteria [17]. However, these guidelines are difficult to apply for children considering the challenges in executing pulmonary function tests and the scarce availability of provocation tests. Therefore, suggestive history, respiratory symptoms, and radiologic tests (Rx, HRCT) represent the initial fundamental assessment to diagnose childhood HP. Laboratory tests and pulmonary function tests, when possible, can further guide the diagnosis. The evaluation of the response after antigen avoidance is also helpful. In uncertain cases, bronchoscopy with BAL should be performed and, in selected cases, the provocation test (Fig. 1, Table 3). An early diagnosis is crucial in children [43, 44]. In case of delayed diagnosis and treatment, HP can convert into a chronic and progressive form, and it can also be fatal (29% of cases at 5 years) [44–47].

**Table 3:** Diagnostic criteria for HP in children (modified from [28])

Suggestive characteristics for HP: 1. Known exposure history to trigger antigen a. positive aerobiological and microbiological environmental investigations b. Sieric specific IgG levels [precipitins, ELISA, ImmunoCAP] 2. Compatible HRCT patterns^a^ 3. Lymphocytosis at BAL (aspecific, not always necessary) 4. Positive inhalation challenge (only in selected patients) • Environmental restatement • Provocation test to the antigen	CONFIDENT HP without biopsy: - Criteria 1 + 2 + 3: Histopathologic confirmation not necessary - Criteria 1 + 2: BAL not necessary in case of clear exposition, typical HRCT, and response after antigen avoidance PROBABLE HP^b^ - Criteria 1 (a or b) + 3; HRCT consistent with other lung diseases - Criteria 2 + 3; no environmental exposure or serologic evidence POSSIBLE HP^b^ - Criteria 1 (a or b); HRCT consistent with other lung diseases; BAL not performed or without lymphocytosis - Criteria 1 (a or b) + 2; BAL without lymphocytosis

^a^HRCT patterns in acute HP: little centrilobular nodules predominantly in upper and middle lobes; ground-glass attenuation; reduced attenuation and vascularization lobular areas. HRCT patterns in chronic HP: upper and middle lobe or peribronchial-vascular fibrosis; honeycomb; air trapping; ground-glass attenuation; centrilobular nodules and relative sparing of the bases

^b^Lung biopsy appropriate for HP confirmation or identification of other diseases

### Anamnesis

A careful environmental history is mandatory for detecting potential triggers [9]. Therefore, specific surveys on the environment should take into account exposures at home, school, and hobbies (e.g., breeding of birds, such as pigeons or parrots; use of wind instruments, water damages at home), agricultural dust, bioaerosols, microorganisms (fungal, bacteria, protozoa) and small molecular weight chemicals (such as plastics, talc) [42, 48]. Of note, only in 17% of pediatric cases, the antigen could not be identified from clinical history [10]. Usually, long-term exposure is required to develop the disease, but cases with occasional, brief exposures have been reported [29].

### Lung imaging

Radiological abnormalities at the chest X-ray help identify disease stage and severity [49, 50]. In the acute form, X-ray ranges from normal to a transient micronodular pattern in the middle and lower zones; in the chronic form, it shows a nodular outline in the middle-upper lung areas*.* Chest High-Resolution Computed Tomography (HRCT) is increasingly used in diagnosing HP; it is more sensitive and offers a more excellent definition of radiographic patterns and information on the presence or absence of fibrosis [51]. The radiological picture varies according to the stage of the disease. In the acute phase of HP, HRCT typically shows ground-glass opacification or diffuse consolidation or patchy air-space; however, it may be normal due to the fleeting nature of the radiographic opacities. In the chronic phase, small centrilobular nodules and lobular areas of reduced attenuation and vascularization are observed (mosaic attenuation). Air trapping can be demonstrated by comparing the inhaling and exhaling images. Interstitial fibrotic lesions might appear as thickening of the septum, traction bronchiectasis, and honeycomb pattern.

### Laboratory studies

Laboratory data usually show leukocytosis, increased ESR, and increased levels of C-reactive protein (CRP) and immunoglobulins. In addition, peripheral eosinophilia may be present. The detection in the serum of IgG precipitating antibodies (ELISA test, ImmunoCAP, precipitin test in Ouchterlony assay) [52] to the triggering antigen has been widely studied as it has been frequently documented in patients with HP. Specifically, 93% of children from Buchvald’s Danish cohort had precipitating antibodies towards relevant antigens, especially mold and pets [7]. However, precipitins can also be detected in asymptomatic individuals exposed to the particular antigen, and negative results do not exclude the diagnosis. Moreover, false-negative results may be due to commercial laboratory kits that test only a small fraction of the potential antigen [51]. In vitro lymphocyte proliferation test after exposure to patients’ serum and/or bronchoalveolar lavage is currently considered for research only [53].

### Pulmonary function tests

Pulmonary function tests (PFT) support the diagnosis of HP. However, they are not able to differentiate the different forms of ChILD [54]. Generally, PFTs show a restrictive pattern, characterized by decreased forced vital capacity (FVC) and total lung capacity (TLC). Spirometry can be normal between acute attacks. In the chronic phase, an obstructive pattern can be observed, and DLCO is typically reduced. Diffusion capacity and lung compliance might be reduced. Moreover, resting oxygen saturation can be normal; however, oxygen desaturation with exercise or sleep may be observed. In advanced disease at adult age, resting oxygen desaturation or pulmonary hypertension may occur [55].

### Antigen identification

It is essential to identify the responsible antigen. Ideally, a provocation challenge represents the diagnostic gold standard [56]. Inhalation of the putative antigen follows an avoidance of at least 72 h with environmental re-exposure or controlled laboratory nebulization and monitoring of symptoms and PFTs in the following 24 h. However, this technique is rarely used in children and is usually limited to research purposes [57].

### Bronchoscopy, bronchoalveolar lavage (BAL) and lung biopsy

Bronchoalveolar lavage (BAL) is the most sensitive tool for detecting alveolitis in patients with suspected HP and appears to reflect the distribution of cell populations shown in biopsies [58, 59]. BAL in children with HP usually has the same characteristics as adults with HP [41]. However, it is not always necessary, particularly among patients with a clear exposure history and typical CT findings (Table 3). In addition, BAL results are nonspecific and may be seen in asymptomatic individuals with antigen exposure or individuals with nonspecific interstitial pneumonia. A marked lymphocytosis CD8+ >  20% and often > 50% of the recovered white blood cells (WBCs) is nonspecific but valuable in the case of suggestive HP [41]. A pulmonary biopsy is rarely needed and usually reserved for cases without adequate clinical criteria for HP. Non-caseous granulomas with an accumulation of multinucleated giant cells and patchy peribronchial infiltrates with lymphocytes have been observed [59, 60].

## Diagnostic algorithm

HP should be suspected in patients with an identified exposure to a triggering antigen and among those with respiratory symptoms and imaging signs of interstitial lung disease without specific characteristics of other diseases (e.g., cystic fibrosis) [2]. BAL is not necessary in case of known exposure history to a specific trigger, typical HRCT, and response to treatment [28]. According to the presence of the leading HP characteristics, “confident”, “probable”, and “possible” diagnosis could be assessed (Table 3). The option of confirming the diagnosis using histopathology is individualized in cases of insufficient criteria. In selected patients, an inhalation challenge could be useful [40]. However, the most representative diagnostic test is imaging with HRCT (Fig. 1). Diffuse ground-glass opacity and hyperdensity are the most significant radiological signs [41]; 96% of the HRCT scans showed characteristic nodular opacities, 5% linear opacities, and 73% ground-glass pattern with increased attenuation [44]. In the study of Buchvald et al., all 19 patients had abnormal HRCT at the time of diagnosis with a diffusely increased attenuation of the parenchyma [7]. PFTs support the diagnostic work-up. All children of the Danish cohort had severely restricted lung function at diagnosis (average FVC 38% of predicted), reduced diffusion capacity for CO, and marked desaturation on exercise [9]. Also, in the French cohort, all children who could perform the lung function test (9/16) had restricted lung function at diagnosis (mean total lung capacity 59% ± 6% of predicted values), and six children had reduced diffusion capacity for CO with a mean of 62% ± 44% of predicted values [41]. The serum precipitin level was relevant for 11 French children [41]. In contrast to the type of exposure obtained from history, 15 children had elevated IgG antibodies against fungi and birds or downy feathers. Only 5 of the 15 children with a history suggestive of a reaction against birds or downy feathers had only these antibodies, and only 1 of the 3 with a history suggestive of fungi reaction had antibodies solely against fungi. Thus, the specificity of elevated IgG antibodies is very low [9]. It is essential to underline that invasive tests are unnecessary if the child is in good general condition or is stable, and the treatment can be started, as previously described for ILD [43]. Bronchoscopy and bronchoalveolar lavage were performed in 12 children of the French cohort [41]. Alveolitis was always present, with average cellularity of 637,000 cells/mL and an average rate of lymphocytes of 37.4% (with considerable variation, ranging from 5 to 94%). The average lymphocyte rate for subacute disease was 34% [41]. Total cell count was elevated in some but not all cases, whereas the cell differential showed a lymphocytosis in 91% (21 of 23) [9]. Lung biopsy was performed only for three children in the French cohort and was performed several years after their diagnosis. The lung biopsy was usually performed due to the chronic evolution with an insufficient response to the treatment [41]. In the German cohort, only six children out of 22 patients needed lung biopsy [7]. BAL was performed in the Danish cohort of children with HP, mainly to rule out infection, but unfortunately, routine cell counts were not performed [10]. Of the 86 reported pediatric cases of HP, 10 biopsy specimens were obtained, and all showed typical histologic changes [40]. A positive provocation test has been reported by Fan and colleagues in 11/20 tested children [40]. It has not been reported in the other cohorts [7, 10, 41].

## Differential diagnosis

Several diseases mimic HP and should be considered in the differential diagnosis of both acute and chronic HP (Table 4). Regarding acute HP, acute viral or atypical bacterial respiratory infection and asthma exacerbation are the primary diseases that should be considered in the differential diagnosis. Unlike these conditions, acute HP is characterized by persistent symptoms despite antibiotic therapy and symptomatic inhalation treatment, the spontaneous resolution after the environment change, and recurrence after a new exposure to the antigen [58]. Asthma can also be considered in the differential diagnosis of chronic HP. Specifically, a positive history for an environmental trigger and HRCT are helpful in differential diagnosis [58]. In addition, predisposing systemic disorders, such as cystic fibrosis, congenital heart disease, immunodeficiency, autoimmune and metabolic diseases, sarcoidosis, vasculitis, should be ruled out with specific diagnostic tests (Table 4).

**Table 4:** Differential diagnosis: predisposing systemic disorders

Sweat test	Cystic fibrosis
Cardiological evaluation (ECG, echocardiogram)	Congenital heart disease
Cultures or tests for infectious aetiology	Lung infections
Oesophagal transit X-ray, pH-impedancemetry	Recurrent aspiration (GERD, dysphagia, anatomical abnormalities)
*Immunity studies* IgA-M-G; recall Ag; HIV Lymphocyte subpopulations; Complement	Immunodeficiencies
*Studies for connective tissue diseases* Anti-nuclear antibodies (ANA) Angiotensin-converting enzyme (ACE) Anti-neutrophil cytoplasmic (ANCA) Anti-glomerular basement membrane (GBM)	Autoimmune diseases Sarcoidosis Vasculitis (e.g., Wegener syndrome, Churg-Strauss syndrome, microscopic polyangiitis) Anti-GBM syndrome (Goodpasture syndrome)
Serum and urinary amino acids	Lysosomal storage diseases Protein intolerance with lysinuria
Genetics for surfactant dysfunctional diseases	Deficit of surfactant production and metabolism
Bronchoscopy, BAL and lung biopsy	Infections, aspiration, Langerhans cell histiocytosis, alveolar haemorrhage, pulmonary alveolar proteinosis

## Prognosis

The prognosis of childhood HP is generally good when the culprit antigen is removed from the child’s environment [7, 10, 40, 58]. Interestingly, in the series reported by Fan et al. [40], approximately 97% of children affected by HP improved, 1.5% got worse, and 1.5% died. Corticosteroids are useful to lead to symptom resolution and improvement of pulmonary function [40, 58]. An in-depth study of lung function in children affected by HP demonstrated that the long-term prognosis is good, although persistent peripheral airway involvement can be present in about half of the subjects [55]. Specifically, the patients with an abnormal FEV1, lung clearance index, and DLCO were 9, 47, and 41%, respectively. In addition, 11% of children demonstrated abnormal maximal oxygen uptake during standardized exercise testing. However, spirometric parameters and DLCO did not change between the end of treatment and the follow-up time [55]. On the other hand, when the diagnosis is delayed, the prognosis is characterized by progressive pulmonary fibrosis and chronic severe lung disease [10].

The genetic and environmental risk factors can influence HP prognosis [61]. Specifically, gene variants within the major histocompatibility complex are related to increased susceptibility to HP, especially fibrotic HP. In addition, the altered expression of MUC5B and genes coding protein-altering telomere-related gene variants, such as TERT, RTEL, and PARN, can have a role. On the other hand, previous respiratory tract infections caused by Epstein-Barr virus, human herpesvirus 7 and 8, cytomegalovirus, parvovirus 19, and exposure to organochlorine and carbamate pesticides has been associated with HP.

## Treatment

Trigger avoidance is the most effective HP treatment, and it usually determines its regression [62]. The recurrence risk is unclear. Trigger avoidance may be simple, such as removing bird bedding or feathers, avoiding hot tubs, or sterilizing humidifiers. However, complete avoidance may sometimes require drastic changes as moving to a new house. Of note, high levels of avian antigens can be detected in the domestic environment for a long time. Nevertheless, not all cases resolve after the avoidance of the antigen involved.

In these instances, the use of corticosteroids can be helpful to lead symptoms resolution and improvement of pulmonary function [40, 58]. Regarding steroid therapy, oral prednisone can be considered a good treatment option. Spagnolo et al. suggested a short cycle of oral prednisone (0.5-1 mg/kg per day) up to 2-3 weeks in acute HP [32]. On the other hand, chronic HP should be treated with higher doses and a longer duration of steroid administration (starting from an initial dose of oral prednisone 0.5 mg/kg body weight per day for a period of 4-to-8 weeks followed by 3 months of decalage) [32]. Intravenous steroids should be considered in case of persistent symptoms and severe impairment in pulmonary function studies and HRCT [40]. Intravenous methylprednisolone pulse therapy using 10 mg/kg per day was often chosen in previous studies [7]. However, the lack of randomized controlled trials in children makes the role of systemic glucocorticoid therapy unclear. In addition, no evidence-based guidelines are available [44]. Consequently, childhood interstitial lung disease (chILD) guidelines represent the reference, especially in children ventilated or closed to ventilation. Specifically, for these selected cases, a combination therapy with intravenous methylprednisolone, hydroxychloroquine, and azithromycin should be considered [43]. Based on these guidelines, other off-label therapies are available [43]. Single cases in adulthood treated with off-label immunosuppressants, such as azathioprine or mycophenolate, have been reported [63, 64]. Clinical trials are needed to understand their effectiveness. Experimental trials are going on rituximab, a monoclonal antibody that depletes B cells, used in individual cases of refractory pneumonia [65]. In addition, the antifibrotic agent nintedanib slows the progression of idiopathic pulmonary fibrosis (IPF) and interstitial lung disease associated with systemic sclerosis [66]. Inhaled steroids can have a role, although trials are needed to evaluate this approach [63, 67]. Supportive therapy includes the discontinuation of exposure to smoke and irritants, nutritional support, oxygen therapy in case of desaturation, respiratory physiotherapy, bronchodilators for reversible obstruction, aggressive treatment of intercurrent infections, vaccinations including influenza and RSV immunoprophylaxis.

## Conclusions

The diagnosis of HP should be considered in any child presenting chronic/recurrent cough or exertion dyspnea with a restrictive pattern on PFTs. HP should be suspected in case of known exposure to HP causative agents and among patients with clinical and imaging evidence of nonspecific interstitial disease. Considering the peculiarity of HP in children, many pediatricians are not familiar with the clinical presentation and diagnosis of this disease. Suggestive history, respiratory symptoms, and radiologic tests (Rx, HRCT) represent the initial fundamental assessment to diagnose childhood HP. Laboratory tests and pulmonary function tests, when possible, can further guide the diagnosis. The diagnostic process must evaluate the exposure with an in-depth environmental survey, assess the severity of the respiratory impairment, and identify the imaging features. When the diagnosis remains unclear, a bronchoscopy and, in selected cases, a provocation test can help in the diagnostic work-up. The prognosis of childhood HP is generally good when the involved antigen is removed from the environment. The role of systemic glucocorticoid therapy is unclear. Trials on treatment are needed, particularly on biological drugs.

## Data Availability

Not applicable.

## Abbreviations

BAL: Bronchoalveolar Lavage
chILD: Childhood interstitial lung disease
CM: Cow’s milk
CRP: C-reactive protein
EAACI: European Academy of Allergy and Clinical Immunology
EAA: Extrinsic allergic alveolitis
FVC: Forced vital capacity
HP: Hypersensitivity pneumonitis
HRCT: High-Resolution Computed Tomography
IPF: Idiopathic pulmonary fibrosis
PFT: Pulmonary function tests
TLC: Total lung capacity
WBCs: White blood cells
